# Food-Grade Metal–Phenolic Network and Polysaccharide Microencapsulation Enhances the In Vivo Anti-Colitic Efficacy of *Lactiplantibacillus plantarum* B112

**DOI:** 10.3390/nu18203422

**Published:** 2026-10-19

**Authors:** Zhenyu Wang, Hongsen Yu, Yuwei Zhang, Ruobing Zhou, Chun Xue, Yixin Jin, Juan Wei, Xiaoqun Zeng, Dao-Dong Pan

**Affiliations:** 1 State Key Laboratory for Quality and Safety of Agro-Products, Ningbo University, Ningbo 315211, China; wzy00125@163.com (Z.W.); yhsen1219@163.com (H.Y.); daodongpan@163.com (D.-D.P.); 2 College of Food Science and Engineering, Ningbo University, Ningbo 315800, China; zhangyuwei0527@163.com (Y.Z.); 19511206176@163.com (R.Z.); weijuan0923@163.com (J.W.); 3 Key Laboratory for Food Microbiology and Nutrition of Zhejiang Province, Ningbo University, Ningbo 315211, China; 4 Zhejiang-Malaysia Joint Research Laboratory for Agricultural Product Processing and Nutrition, Ningbo University, Ningbo 315800, China; 5 Institute of One Health Science, Ningbo University, Ningbo 315211, China

**Keywords:** probiotics, inflammatory bowel disease, microencapsulation, metal–phenolic network, polysaccharides

## Abstract

Background/Objectives: Probiotic therapy for inflammatory bowel disease (IBD) has a persistent practical obstacle: most cells given by mouth die before they reach the colon. Methods: To keep *L. plantarum (Lactiplantibacillus plantarum*) B112 alive along this route, we first assembled a gallic acid–ferric ion metal–phenolic network (MPN) around the bacteria and then added an outer layer of sodium alginate or hyaluronic acid; the resulting microcapsules, assembled entirely from food-grade materials, were evaluated in mice during a 7-day dextran sulfate sodium (DSS) course. Results: Among all formulations, the alginate-coated ones (LLM@SA; 10^8^ colony-forming units (CFU)/day for 5 days) were the most effective. Body weight loss slowed, the disease activity index fell, and colon shortening was less severe than in the DSS controls. In colonic tissue, interleukin (IL)-1β, IL-6, tumor necrosis factor (TNF)-α, and myeloperoxidase decreased, IL-10 increased, the mucus layer recovered, and mucin 2 (MUC2), zonula occludens-1 (ZO-1), and occludin were maintained; serum lipopolysaccharide (LPS), D-lactic acid (D-LA), and diamine oxidase (DAO) also dropped. 16S rRNA gene sequencing revealed a partial correction of DSS-induced dysbiosis, with *Akkermansia* and *Alistipes* enriched while *Escherichia-Shigella* and *Enterococcus* were suppressed. Conclusions: A plausible interpretation is that the double coating improved the survival of B112 during gastrointestinal transit and allowed more viable cells to reach the large intestine, where the strain could modulate immunity, barrier integrity, and the microbiota. However, viable B112 was not directly quantified in fecal or intestinal samples, and coating-only control groups were not included, so this mechanism remains inferential and a contribution of the coating materials themselves cannot be excluded. The data therefore support further preclinical investigation of this microencapsulation strategy, assembled from food-compatible materials, as a nutritional approach in models of IBD.

## 1. Introduction

Inflammatory bowel disease (IBD), comprising mainly ulcerative colitis (UC) and Crohn’s disease (CD), affects approximately 6.8 million people worldwide and generally follows a chronic course accompanied by repeated relapses. In most patients, persistent disease is driven mainly by intestinal mucosal inflammation that is not resolved in time, and this inflammation is typically accompanied by dysbiosis of the resident gut microbiota, weakening of the intestinal epithelial barrier, and disordered mucosal immune responses [1,2,3,4]. Current mainstream treatment relies mainly on aminosalicylates, corticosteroids, immunomodulators, and biologics [5,6,7]. When these therapies are administered alone, they in most cases provide only short-term relief of symptoms and cannot effectively improve patients’ health over the long term. Moreover, adverse effects that may arise during long-term treatment can force clinicians to reduce the drug dose, or even interrupt therapy, when symptoms relapse repeatedly. Given the predicament of very limited adjunctive options, dietary interventions capable of remodeling the gut microbiota have gradually come into view [8].

Among these diet-related interventions, probiotics occupy a prominent position. A probiotic is generally regarded as an ingestible microorganism that exerts positive effects on the host and typically helps to maintain microbial balance within the host intestine. In IBD, the actions of probiotics mainly include maintaining the homeostasis of the gut microbiota, strengthening the tight junctions between intestinal epithelial cells, and exerting a degree of inhibition on intestinal mucosal inflammation, yet the clinical evidence assembled to date remains inconsistent [9,10]. In addition, whether probiotics can exert the above actions depends mainly on whether live cells are able to reach the distal intestine, a prerequisite that still faces many difficulties and challenges in clinical practice [11]. First, heat and oxygen exposure arising during both the production process and cold-chain storage of probiotic products negatively affects probiotic survival. The bacteria that survive must then confront the challenges of bile salts and the strongly acidic gastric environment (pH 1.5–3.5), and in the course of overcoming these hurdles, every step further reduces the number of viable cells that ultimately reach the target site [12,13]. Consequently, the benefit a probiotic can provide is likely to depend not only on the intrinsic properties of the strain but also on its delivery capacity.

Attacking this delivery problem is the point of departure for the present work. In an earlier report from our group [14], *Lactiplantibacillus plantarum* B112 (China General Microbiological Culture Collection Center [CGMCC] No. 34013) carrying a metal–phenolic network (MPN)–polysaccharide double layer withstood heat, ultraviolet irradiation, lyophilization, and simulated gastrointestinal transit far better than uncoated cells. As expected for this species, the strain retained traits commonly associated with *L. plantarum*, including stress tolerance and potential immunomodulatory properties. Unprotected cells, the same study showed, lost viability rapidly under simulated upper-gastrointestinal conditions, implying that most orally administered bacteria would die before reaching the colon. Whether these in vitro advantages translate into protection in a living animal had not been examined.

Wrapping bacteria in a protective shell to improve stress resistance and delivery is a long-established strategy [12]. More recently, metal–phenolic networks (MPNs), built by coordination between phenolic ligands and transition-metal ions, have been explored as single-cell coatings for food applications [15]; such coatings shield probiotics against ultraviolet irradiation, oxidative damage, and freeze-drying and can promote intestinal colonization [13]. They have a recognized drawback, though: in the acidic stomach, protonation of the hydroxyl groups on phenolic rings weakens metal–polyphenol coordination and the shell disintegrates [15]. With this limitation in mind, we constructed a food-grade microcapsule in which gallic acid and ferric ions formed an MPN around B112, and the coated cells were then wrapped in sodium alginate or hyaluronic acid [14]. That earlier study reported improved encapsulation stability, environmental tolerance, and in vitro gastrointestinal protection, but left two questions open: whether the benefit would survive in an IBD animal model, and how the microcapsules would behave in vivo toward immunity, barrier function, and the microbiota.

The present work addresses these questions in mice with dextran sulfate sodium (DSS)-induced colitis. Unencapsulated B112 and three coated preparations were given daily by gavage on the premise that the double layer would carry a larger share of the dose through the gastrointestinal tract to the colon. Efficacy was followed with complementary readouts covering body weight, the disease activity index, colon length, histopathology, organ indices, inflammatory cytokines, and myeloperoxidase, together with barrier function and the fecal 16S rRNA gene profile. In this way the study links our earlier in vitro observations to whole-animal efficacy and asks whether food-grade microencapsulation merits further attention as a nutritional adjunct in the management of IBD.

## 2. Materials and Methods

### 2.1. Materials

*Lactiplantibacillus plantarum* B112 (CGMCC No. 34013), the probiotic strain used throughout this work, came from the Institute of Animal Science and Food Processing, Ningbo University. The strain is deposited at the China General Microbiological Culture Collection Center (CGMCC No. 34013), and its stress resistance and survival under simulated gastrointestinal conditions were characterized in our previous work [14]. MP Biomedicals, LLC (Santa Ana, CA, USA) supplied the DSS (36–50 kDa), while gallic acid (GA), ferric chloride hexahydrate, sodium alginate (SA), and hyaluronic acid (HA) were obtained from Macklin Biochemical Co., Ltd. (Shanghai, China). The enzyme-linked immunosorbent assay (ELISA) kits for interleukin (IL)-1β, IL-6, tumor necrosis factor (TNF)-α, IL-10, lipopolysaccharide (LPS), D-lactic acid (D-LA), and diamine oxidase (DAO) were provided by Shanghai Enzyme-linked Biotechnology Co., Ltd. (Shanghai, China). Every other chemical was of analytical grade and was used as received.

### 2.2. Preparation of Probiotic Microcapsules

Cultivation of *L. plantarum* B112 (CGMCC No. 34013) followed our earlier protocol [14] apart from small adjustments made for the animal trial. Revival of the frozen stock took place on MRS agar (de Man, Rogosa and Sharpe), and two further subcultures in MRS broth brought the culture to the stationary phase after 12 h at 37 °C. Harvested pellets were washed with phosphate-buffered saline (PBS, pH 7.4) and finally resuspended at 10^9^ colony-forming units (CFU)/mL. Assembly of the MPN shell began with the successive addition of GA (1 mg/mL) and FeCl_3_·6H_2_O (0.6 mg/mL); coordination was allowed to proceed while the suspension was held at 37 °C [14]. The MPN-coated *L. plantarum* B112 (LLM) pellets obtained in this way were then suspended in alginate or hyaluronic acid (5 mg/mL; SA and HA), incubated with gentle shaking at 37 °C for 30 min, and pelleted by centrifugation to strip unbound polysaccharide, which yielded the final LLM@SA (alginate-coated) and LLM@HA (hyaluronic acid-coated) particles used in the trial. Plating on MRS agar (standard plate count, SPC) served to enumerate free and encapsulated cells, and survival across lyophilization was calculated from the viable cell count after the process divided by the count before it. Gallic acid, ferric chloride hexahydrate, sodium alginate, and hyaluronic acid were of food-grade or analytical purity. We note, however, that the grade of the individual reagents does not by itself establish that the final microcapsule formulation is food-grade or suitable for human consumption, and no safety assessment for human use was performed in this study.

### 2.3. Animal Experiment Design

**Figure 1:**
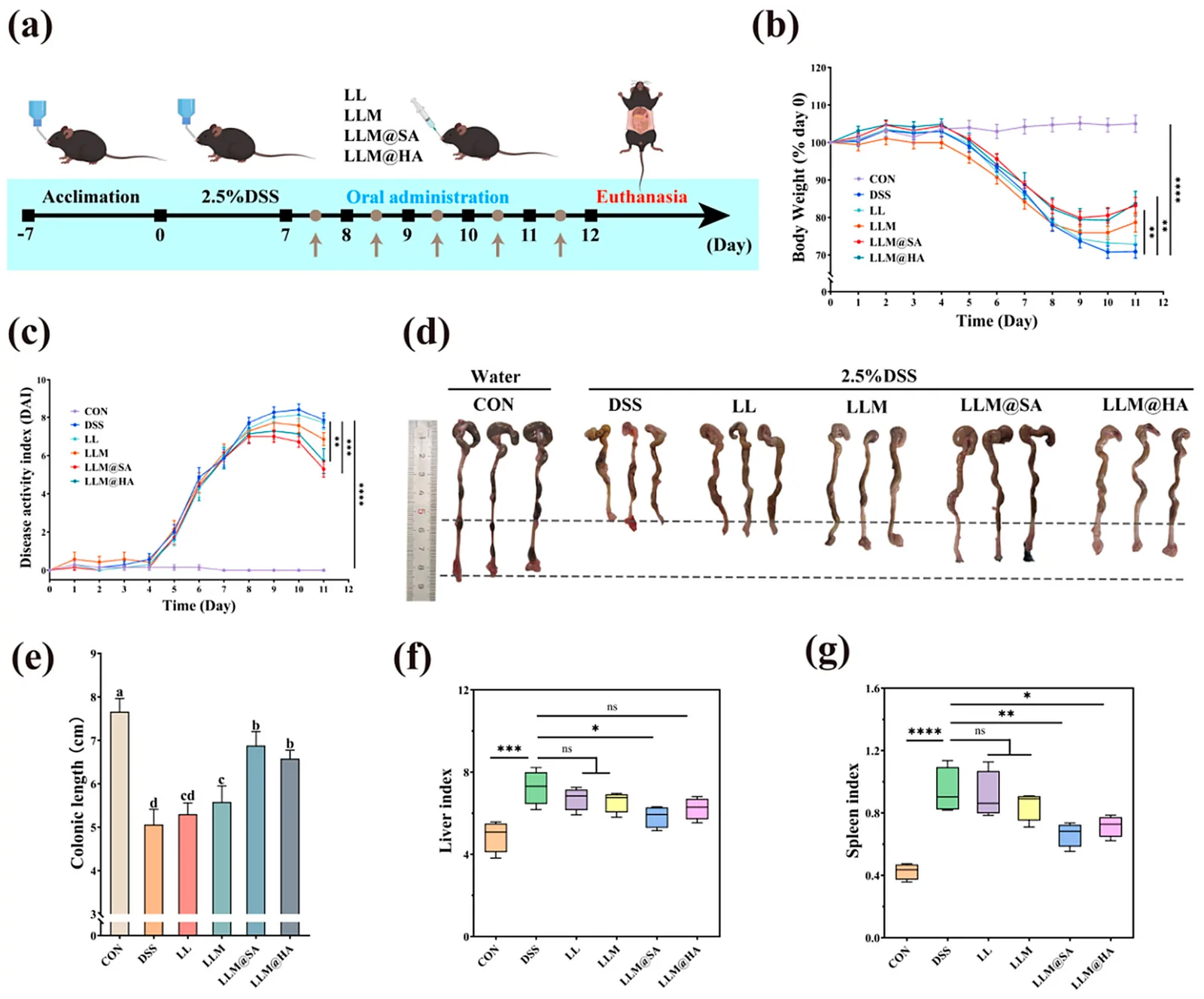
*Lactiplantibacillus plantarum* B112 microcapsules alleviate DSS-induced colitis symptoms in mice. (**a**) Schematic illustration of the animal experimental design and treatment schedule. (**b**) Body weight changes in mice during DSS induction and oral administration. (**c**) Disease activity index (DAI) changes in mice during the experimental period. (**d**) Representative photographs of colons from mice in different treatment groups. (**e**) Quantitative analysis of colon length in different groups. (**f**) Liver index of mice after different treatments. (**g**) Spleen index of mice after different treatments. Data are presented as mean ± SD (n = 7). * *p* < 0.05, ** *p* < 0.01, *** *p* < 0.001, and **** *p* < 0.0001; ns, not significant. Bars labeled with different lowercase letters differ significantly (*p* < 0.05). The arrows in (**a**) indicate the daily gavage period.

Figure 1a outlines the experimental design. Beijing Vital River Laboratory Animal Technology Co., Ltd. (Beijing, China) supplied the male C57BL/6J mice (specific pathogen-free [SPF], 6–8 weeks old), which were kept in the Laboratory Animal Center of Ningbo University, also under SPF conditions. All mice were housed in the same SPF facility under identical environmental conditions throughout the experiment. The animals were allowed 1 week to acclimate (food and water ad libitum; 22 ± 2 °C and 50 ± 10% relative humidity; 12 h light/dark cycle) before being divided into six groups of seven, namely CON (normal control), DSS (colitis model), LL (unencapsulated *L. plantarum* B112), LLM (MPN-coated), LLM@SA (alginate-overcoated), and LLM@HA (hyaluronic acid-overcoated). No formal a priori sample-size or power calculation was performed; the group size (*n* = 7) was chosen on the basis of our laboratory’s prior experience with this DSS colitis model and of group sizes commonly used in comparable studies [16]. Colitis was induced by replacing the drinking water with 2.5% DSS ad libitum for 7 days in every group except CON, after which plain water was restored. From day 8 onward, gavage of the corresponding bacterial suspension (10^8^ CFU/day) was performed once daily for 5 days in the four treatment groups, while CON and DSS mice received an equal volume of PBS. The dose of 10^8^ CFU/day was selected on the basis of our previous work with this encapsulation system [14] and of doses commonly used in probiotic-intervention studies in murine colitis models [17,18]. Viable counts of free and encapsulated cells were enumerated by standard plate counting, and the administered doses were prepared on the basis of these counts. Daily records were kept of body weight and the disease activity index (DAI, scored according to the criteria in Table S1). On day 12 the animals were euthanized in accordance with the approved protocol after blood collection from the retro-orbital plexus; serum was prepared, and colons were excised and measured. A distal colon segment was taken, placed in 4% paraformaldehyde solution for fixation, and used for histological examination, while the remaining tissue was stored at −80 °C. All experimental procedures were approved in advance by the Ningbo University Animal Ethics Committee (Approval No. NBU20250404), and these procedures also conformed to the Chinese national guidelines for the welfare and ethical use of laboratory animals; the experimental reporting strictly followed the Animal Research: Reporting of In Vivo Experiments (ARRIVE) guidelines.

### 2.4. Histopathological Analysis

Colon segments that had been fixed in 4% paraformaldehyde for at least 24 h were dehydrated through an ascending ethanol series, embedded in paraffin, and sectioned at 4 μm. The sections were stained with hematoxylin–eosin (H&E) or with Alcian blue and periodic acid-Schiff (AB-PAS). H&E sections served for histopathological evaluation and scoring (Table S2), and AB-PAS sections for assessment of goblet cell density and mucus layer morphology. Staining was carried out by Wuhan Servicebio Technology Co., Ltd. (Wuhan, China), and the scores were assigned by a pathologist blinded to the treatment groups. For each section, the final histological score was assigned by the blinded pathologist based on a comprehensive evaluation of the four components listed in Table S2.

### 2.5. Immunohistochemistry (IHC) Analysis

After deparaffinization and rehydration, colon sections underwent antigen retrieval and blocking, then were incubated with primary antibodies to myeloperoxidase (MPO), zonula occludens-1 (ZO-1), and occludin, and subsequently with the appropriate secondary antibodies. Signals were developed with 3,3′-diaminobenzidine (DAB), and nuclei were counterstained with hematoxylin. Primary and secondary antibodies and the DAB reagent were provided by Wuhan Servicebio Technology Co., Ltd. (Wuhan, China).

### 2.6. Enzyme-Linked Immunosorbent Assay (ELISA)

IL-1β, TNF-α, IL-6, and IL-10 in colon homogenates were measured with commercial ELISA kits, following the manufacturer’s protocols exactly. Serum LPS, D-LA, and DAO were also quantified with these kits as markers of intestinal permeability.

### 2.7. Reverse Transcription Quantitative PCR (RT-qPCR) Analysis

RT-qPCR was used to quantify colonic mRNA of the barrier genes ZO-1, Occludin, and mucin 2 (MUC2). Total RNA was extracted with a kit from Vazyme Biotech Co., Ltd. (Nanjing, China) and converted to cDNA by reverse transcription. Amplification ran on a Roche LightCycler^®^ 480 (Roche Diagnostics, Basel, Switzerland) with the primers listed in Table S3 and a commercial qPCR kit; Gapdh was the reference gene. Relative expression was computed by the 2^−ΔΔCt^ method.

### 2.8. 16S rRNA Gene Sequencing and Microbiota Analysis

At the end of the experiment, feces were collected from CON, DSS, and LLM@SA mice, snap-frozen in liquid nitrogen, and stored at −80 °C. Twelve samples in total, four per group, were submitted for 16S rRNA gene amplicon sequencing. DNA was isolated from each sample, and the V4 hypervariable region was PCR-amplified with primers 515F/806R. Library preparation and sequencing used an Illumina NovaSeq 6000 instrument (Illumina, San Diego, CA, USA). After merging and quality filtering of the reads and removal of chimeras, amplicon sequence variants (ASVs) were resolved with DADA2 (https://benjjneb.github.io/dada2/, accessed on 12 October 2026) under QIIME2 (https://qiime2.org, accessed on 12 October 2026) and classified against SILVA 138.1. Library construction, sequencing, and bioinformatics were outsourced to Novogene Co., Ltd (Beijing, China). Fecal samples from four of the seven mice per group were used for sequencing, and the analysis was restricted to the CON, DSS, and LLM@SA groups, which constitute the core comparison among the healthy control, the disease model, and the most effective formulation; the selection of animals for sequencing was not pre-specified. The raw 16S rRNA gene sequencing data have been deposited in the NCBI Sequence Read Archive (SRA) under BioProject accession number PRJNA1541253.

### 2.9. Statistical Analysis

Results are reported as means ± standard deviation (SD). Group comparisons relied on one-way analysis of variance (ANOVA) followed by Duncan’s multiple range test in SPSS 27.0 (SPSS Inc., Chicago, IL, USA); means that did not share a letter were considered significantly different (*p* < 0.05). Body weight and DAI, which were recorded daily throughout the experimental period, were compared among groups at the final time point. The threshold for significance was *p* < 0.05. Graphs were made with GraphPad Prism 10.1.2 (GraphPad Software, San Diego, CA, USA) and ChiPlot (https://www.chiplot.online, accessed on 12 October 2026). Beta diversity was evaluated by principal coordinate analysis (PCoA) and non-metric multidimensional scaling (NMDS) based on Bray–Curtis and weighted/unweighted UniFrac distances, and the ordination plots were interpreted visually.

## 3. Results and Discussion

### 3.1. Encapsulated L. plantarum B112 Attenuated DSS-Induced Colitis Symptoms

The capsules were evaluated in male C57BL/6J mice subjected to the 7-day DSS regimen, a standard model recapitulating key features of human IBD, including disruption of the epithelial barrier, bacterial translocation, and intestinal inflammation [16]. DAI values recorded before gavage were similar across the DSS-treated groups (*p* > 0.05), indicating that colitis of comparable severity had developed in every arm.

Body weight responded to both the severity of colitis and the effect of treatment. Once gavage began on day 8, weight loss slowed in every intervention group compared with the DSS controls. LLM@SA showed the best course, with body weight starting to rise on the third day of treatment, one day earlier than in the other groups (Figure 1b). Uncoated B112 (LL) did little better than DSS, as would be expected if most unprotected cells do not survive gastric passage. The order of efficacy (LL < LLM < LLM@HA < LLM@SA) is compatible with, but does not demonstrate, improved viable-cell delivery to the gut, since viable B112 was not enumerated in fecal, cecal, or colonic samples.

The disease activity index combines several clinical signs into one score that tracks disease severity. In the DSS-treated groups the score climbed well above the CON level (*p* < 0.05; Figure 1c) and then, in LLM@SA mice, fell markedly after gavage (*p* < 0.05). Colon shortening, another hallmark of acute colitis, was present to different degrees in all model groups (Figure 1d); LLM@SA showed the mildest changes, with colon length near normal and only slight edema. Direct measurement (Figure 1e) placed the LLM@SA group close to the healthy controls, significantly longer than the DSS group (*p* < 0.05).

Inflammation of this kind is usually not confined to the intestine. Immune responses arising elsewhere in the body often spread to distant organs, the liver and kidney among them [17]; we therefore recorded the liver and spleen indices (Figure 1f,g). Compared with CON, the liver index was significantly elevated in both the DSS and LL groups (*p* < 0.05); such elevations are usually caused by inflammation-driven dysbiosis, together with toxic metabolites that alter metabolic gene expression and impair hepatic function. In the LLM@SA-treated mice, this index was significantly lower than that of the DSS group (*p* < 0.05); however, because liver histology and serum transaminase activities were not examined, the lower liver index cannot by itself be taken as evidence of liver injury or of protection from it and is more conservatively interpreted as reflecting a reduced systemic inflammatory burden. The spleen index showed a similar trend, a pattern that reflects the systemic inflammatory load. After DSS induction, the spleen index rose sharply (*p* < 0.05) and declined significantly after LLM@SA treatment; these observations point to an alleviation of the systemic inflammatory burden after treatment.

### 3.2. Lactiplantibacillus plantarum B112 Microcapsules Attenuated Colon Inflammation in DSS-Treated Mice

**Figure 2:**
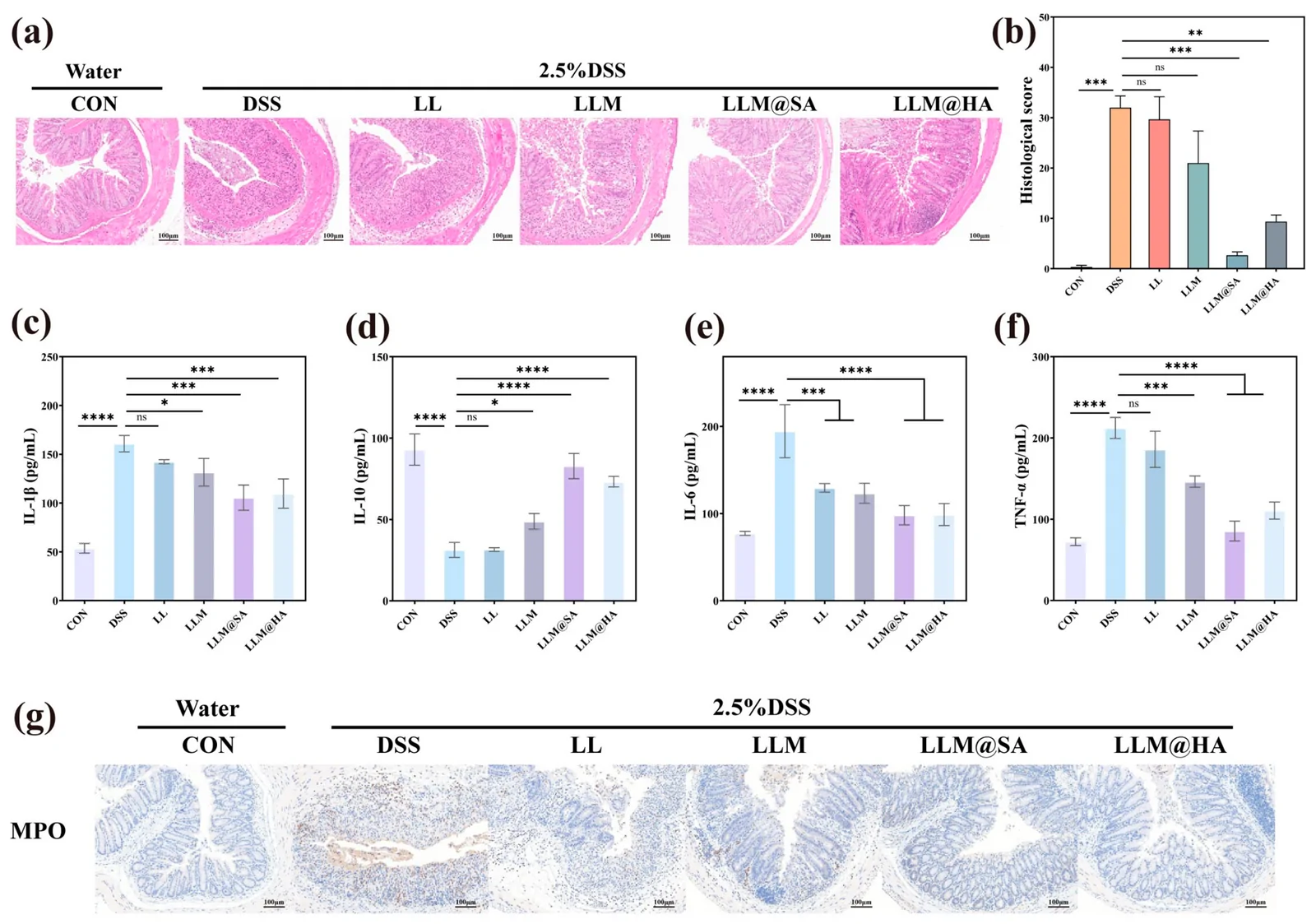
*Lactiplantibacillus plantarum* B112 microcapsules alleviate DSS-induced colonic inflammation in mice. (**a**) Representative H&E-stained images of colon tissues from mice in different treatment groups. (**b**) Histological scores of colon tissues in different groups. (**c**) IL-1β levels in colon tissues. (**d**) IL-10 levels in colon tissues. (**e**) IL-6 levels in colon tissues. (**f**) TNF-α levels in colon tissues. (**g**) Representative immunohistochemical staining images of myeloperoxidase (MPO) in colon tissues. Scale bars in (**a**,**g**): 100 μm. Data are presented as mean ± SD (n = 7). * *p* < 0.05, ** *p* < 0.01, *** *p* < 0.001, and **** *p* < 0.0001; ns, not significant.

Marked differences among the groups were observed when the H&E-stained distal colon sections were examined. We further applied blinded scoring, which comprehensively considered infiltrate density, crypt architecture, and the extent of tissue involvement [18], and found that severe mucosal damage was already present in the colon of the DSS-treated group. The surface epithelium had detached, the crypts had disappeared, leukocytes had infiltrated massively, and goblet cells were almost completely absent. In contrast, the LLM@SA group showed the opposite tendency, with tissue architecture restored almost to the level of the healthy controls (Figure 2a,b).

Histological recovery was most pronounced in the LLM@SA group. Its score fell far below the DSS level, the mucosa showed little difference from a normal appearance, the crypts were compactly arranged, infiltration was reduced, and goblet cells were still present. LLM@HA followed closely, with a significantly lower score and partial restoration of the mucosa and crypt structure, although the mucosal architecture still had not fully returned to normal. The score difference between the LL and LLM groups was very small, and repair in both groups remained incomplete. This pattern would be expected if most administered cells are inactivated during passage through the stomach and proximal intestine: the protection afforded by each shell would then influence how many viable cells reach the colon and, consequently, the extent of subsequent repair. We stress, however, that viable B112 was not enumerated in fecal, cecal, or colonic samples in this study, so this explanation remains inferential, and a contribution of the coating materials themselves cannot be excluded.

Cytokines are key factors in colonic inflammation. TNF-α aggravates tissue injury by promoting the production of reactive oxygen species and inducing necroptosis of epithelial cells, whereas IL-6 maintains the inflammatory cascade by activating T cells and antigen-presenting cells [19,20]. When colon homogenate samples were measured by ELISA (Figure 2c–f), the DSS group, compared with CON, displayed the expected profile: the levels of IL-6, TNF-α, and IL-1β were significantly elevated, whereas the level of IL-10 was reduced. The LLM@SA-treated group showed exactly the opposite situation, with the IL-10 level rising significantly and the levels of the three pro-inflammatory mediators falling back to near the CON level. The anti-inflammatory activity of IL-10 derives mainly from the function of regulatory T cells, which can suppress the production of pro-inflammatory mediators and maintain an environment favorable to mucosal healing [21]. In summary, the cytokine measurements support the anti-inflammatory effect of LLM@SA observed in this study.

Myeloperoxidase (MPO), a marker of inflammatory cell infiltration, is frequently measured in tissues; this enzyme is secreted mainly by neutrophils [22]. To complement the cytokine data, this study applied immunohistochemistry to assess the distribution of MPO in the colon. Both LLM@SA and LLM@HA treatment effectively reduced colonic MPO accumulation (Figure 2g), a finding consistent with the lower degree of local inflammation observed in these two groups.

Comparison of the four treatment arms explains the superior performance of LLM@SA. The LL arm behaved much like the DSS group, presumably because most unprotected administered cells do not survive gastric passage. LLM produced only partial rescue, in line with the tendency of MPN coatings to disintegrate through acid-driven protonation [15]. LLM@SA clearly outperformed LLM, with LLM@HA in between, so the greater efficacy conferred by the alginate over-coat is plausibly related to improved gastric protection and colonic retention of the formulation, although neither was directly demonstrated in vivo. One plausible interpretation is that in the acidic stomach the alginate gel shields the MPN from protonation-induced disassembly, and that in the colon mucoadhesion prolongs retention of the formulation on the inflamed mucosa. However, the present design did not include coating-only (vehicle) control groups, and the polysaccharide layer differs between LLM@SA and LLM@HA; consequently, the superior efficacy of LLM@SA cannot be unequivocally attributed to enhanced delivery of viable B112, as opposed to biological effects of the encapsulation matrix itself (gallic acid, ferric ions, alginate) or to a combination of both. Direct enumeration of viable B112 in cecal or colonic contents, together with coating-only control groups, would be required to resolve this question; the absence of these experiments is a key limitation of the present study.

### 3.3. Lactiplantibacillus plantarum B112 Microcapsules Enhanced Intestinal Barrier Function

A broken intestinal barrier is both a signature of DSS-induced colitis and a prerequisite for bacterial translocation and progressive inflammation. We next asked whether the greater efficacy of LLM@SA was paralleled by repair of the mucus layer, re-expression of tight junction proteins, and normalization of paracellular permeability. Anatomically, the barrier comprises three layers: the mucus gel secreted by goblet cells, the glycocalyx covering the enterocyte surface, and the basolateral junctional complexes—above all the tight junctions and adherens junctions [23,24], which shut the paracellular route. During inflammation, oxidative stress in the lumen injures all three layers, permeability increases, and bacterial endotoxins along with luminal antigens penetrate the lamina propria, aggravating immune-mediated tissue injury [10]. Restoring this multilayered defense is consequently viewed as a central goal in managing IBD.

**Figure 3:**
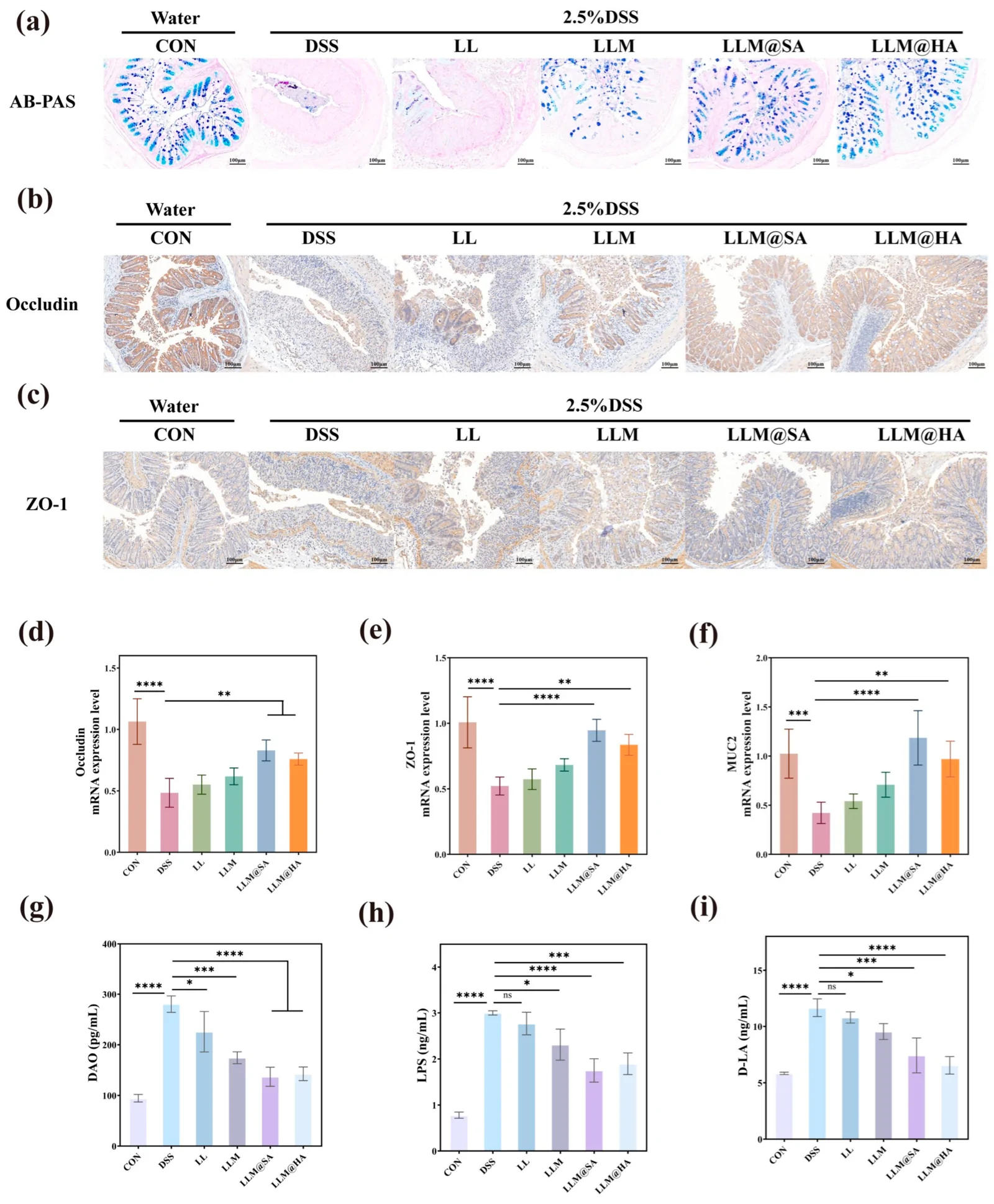
*Lactiplantibacillus plantarum* B112 microcapsules improve intestinal barrier function in DSS-induced colitis mice. (**a**) Representative AB-PAS-stained images of colon tissues from mice in different treatment groups. (**b**) Representative immunohistochemical staining images of Occludin in colon tissues. (**c**) Representative immunohistochemical staining images of ZO-1 in colon tissues. (**d**) Relative mRNA expression level of Occludin in colon tissues. (**e**) Relative mRNA expression level of ZO-1 in colon tissues. (**f**) Relative mRNA expression level of MUC2 in colon tissues. (**g**) Serum diamine oxidase (DAO) levels in different groups. (**h**) Serum lipopolysaccharide (LPS) levels in different groups. (**i**) Serum D-lactic acid (D-LA) levels in different groups. Scale bars in (**a**–**c**): 100 μm. In (**b**,**c**), brown indicates DAB-positive staining and blue indicates hematoxylin-counterstained nuclei. Data are presented as mean ± SD (n = 7). * *p* < 0.05, ** *p* < 0.01, *** *p* < 0.001, and **** *p* < 0.0001; ns, not significant.

The mucus layer and goblet cells were evaluated on AB-PAS-stained sections. In DSS mice the mucus layer had clearly broken down: it was visibly thinner and stained more weakly, with an apparent loss of goblet cells (Figure 3a); goblet cell density was not quantified, so these histological observations are descriptive. A mucus barrier in this condition is thought to facilitate pathogen invasion and the spread of inflammation [25]; in the DSS colon the mucosal and submucosal layers also looked distinctly separated. Some improvement was seen in the LL, LLM, and LLM@HA groups, where goblet cell loss and mucus damage were reduced, yet none of these groups reproduced the morphology of the healthy controls. LLM@SA came closest to recovery, with mucus integrity maintained and goblet cell number and shape near control levels, an indication of partial restoration of the mucus barrier.

Tight junction function depends on occludin and ZO-1 acting together. Occludin stabilizes the junction and controls its dynamics, which preserves barrier integrity and signal transduction; ZO-1, in turn, links transmembrane proteins to the cytoskeleton and keeps the junction organized [26]. Transcript levels of both genes, readouts of tight junction restoration, fell markedly in the DSS group and remained well below the healthy-control values (Figure 3d,e); the corresponding immunohistochemical staining patterns were qualitatively consistent with these changes (Figure 3b,c), although IHC-positive area and staining intensity were not quantified. LL, LLM, and LLM@HA raised these levels to varying degrees, and LLM@SA again gave the highest expression, significantly above the DSS group and each of the other arms. The molecular data thus mirror the barrier-protective effect of LLM@SA.

In the colonic mucus layer, the principal gel-forming mucin is MUC2, a heavily glycosylated protein secreted by goblet cells that provides the first line of defense against harmful agents [27]. Its transcript level in colon tissue was therefore quantified by RT-qPCR. DSS strongly suppressed MUC2 mRNA, whereas LLM@SA raised it significantly (Figure 3f), in line with the AB-PAS findings. The most plausible explanation invokes host–microbe interactions: components and metabolites released by B112 cells present in the gut lumen have been proposed to promote MUC2 transcription and goblet cell secretion [28]; whether the administered B112 persisted in, or colonized, the gut was not assessed in this study.

Intestinal permeability was tracked through the serum markers LPS, D-LA, and DAO [29]. In the DSS group all three were markedly elevated relative to CON (Figure 3g–i), a sign of increased gut permeability. LLM@SA kept them far lower in the bloodstream than LL, LLM, or LLM@HA did, which argues for at least partial repair of the damaged barrier.

Across these endpoints, oral LLM@SA restored the colonic mucosal barrier: tissue injury was alleviated, goblet cell mucus secretion promoted, tight junction protein expression restored, and intestinal permeability reduced. The formulation therefore merits further evaluation in additional preclinical models of colitis.

### 3.4. Lactiplantibacillus plantarum B112 Microcapsules Modulated the Gut Microbiota

Given the superior protection afforded by LLM@SA, we next asked whether the formulation also remodeled the gut microbiota. Fecal 16S rRNA gene amplicon sequencing (V4 region) was consequently used to test whether LLM@SA could attenuate DSS-induced dysbiosis [30].

**Figure 4:**
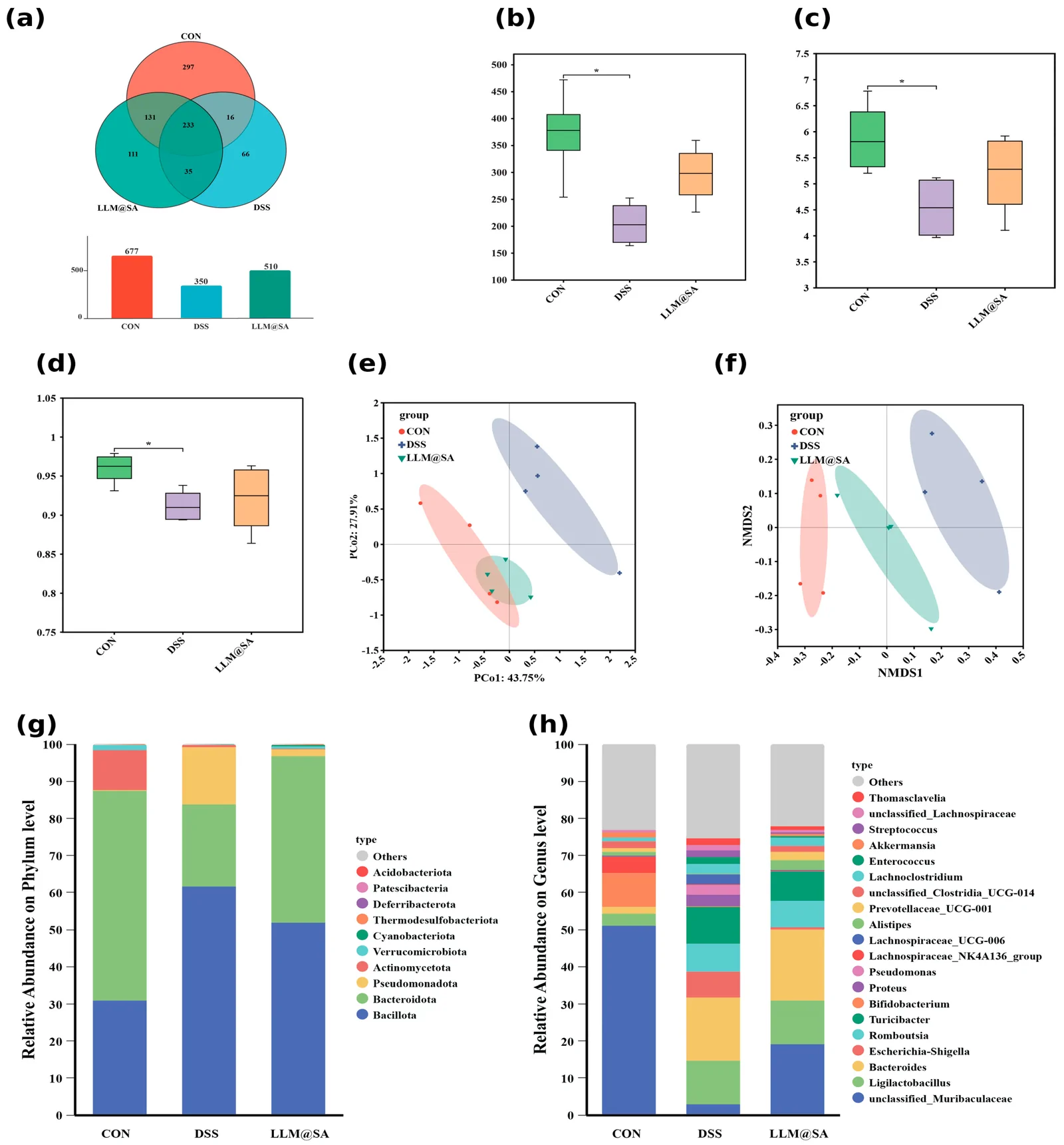
*Lactiplantibacillus plantarum* B112 microcapsules regulate gut microbiota diversity and composition in DSS-induced colitis mice. (**a**) Venn diagram of shared and unique ASVs among different groups. (**b**) Chao1 index of gut microbiota. (**c**) Shannon index of gut microbiota. (**d**) Simpson index of gut microbiota. (**e**) PCoA plot of gut microbiota. (**f**) NMDS plot of gut microbiota. (**g**) Relative abundance of gut microbiota at the phylum level. (**h**) Relative abundance of gut microbiota at the genus level. Data are presented as mean ± SD (n = 7). * *p* < 0.05.

Microbial diversity and richness are widely regarded as indicators of host health, and both typically decline during DSS-induced colitis [31]. The Venn diagram in Figure 4a shows that 233 ASVs were shared among the CON, DSS, and LLM@SA groups, with total ASV numbers of 677, 350, and 510, respectively. Relative to CON, DSS markedly reduced ASV richness, and LLM@SA substantially restored it, indicating that species richness improved after the intervention.

The Chao1, Shannon, and Simpson indices are commonly used to quantify the richness and diversity of the gut microbiota. All three indices decreased significantly after DSS induction (Figure 4b–d), a pattern consistent with the characteristic features of DSS-induced IBD. Treatment with LLM@SA restored these indices to a large extent, suggesting partial recovery of the microbial richness and diversity affected by colitis.

Community-level differences were visualized by PCoA and NMDS ordination (Figure 4e,f). The samples of the CON and DSS groups were located in two clearly separated clusters, consistent with a pronounced shift in community composition caused by colitis. The samples of the LLM@SA group lay closer to the CON group and partly overlapped with it, suggesting that the treatment shifted the microbial community back toward that of the healthy controls; these ordinations were interpreted visually, and no formal multivariate test of community-level differences was performed.

The comparison of taxonomic composition covered the phylum and genus levels (Figure 4g,h). Every group was dominated by Bacillota and Bacteroidota, but with markedly different proportions. DSS reduced the abundance of Bacteroidota, a phylum that includes many carbohydrate-fermenting commensals capable of generating short-chain fatty acids, and simultaneously caused abnormal proliferation of Pseudomonadota (formerly Proteobacteria), a pattern of dysbiosis widely regarded as associated with colitis. After LLM@SA treatment, the microbial balance was partly restored: the abundance of Bacteroidota rebounded, Pseudomonadota was restrained, and the overall microbial composition approached that of CON. The Bacillota/Bacteroidota ratio in the LLM@SA group also showed a downward trend. Shifts in this ratio have been reported in some IBD studies, but its direction and magnitude are highly context-dependent, and it should not be regarded as a standalone biomarker of intestinal health or IBD severity.

The genus level told the same story. Several genera often associated with intestinal homeostasis were strongly depleted by DSS, among them Unclassified_Muribaculaceae, whose members have shown anti-inflammatory associations [32], *Bifidobacterium*, which is associated with a stronger epithelial barrier, greater junction protein expression, and a thicker mucus layer [33], and the short-chain fatty acid producer Lachnospiraceae_NK4A136_group, a contributor to colonization resistance [34]. Abundance meanwhile shifted toward genera carrying inflammatory or pathogenic potential. The steepest rise belonged to *Escherichia-Shigella*, a genus group that includes well-known enteric pathogens; because V4-region 16S rRNA gene sequencing does not provide species- or strain-level resolution, pathogenicity cannot be inferred directly from these assignments. *Enterococcus* also expanded; some members of this genus have been reported to aggravate colitis [35], although genus-level assignments should be interpreted with caution. *Turicibacter* (Erysipelotrichaceae) also increased, a genus reported to become more abundant as pro-inflammatory cytokines rise during DSS colitis [3].

LLM@SA corrected the dysbiosis only in part. Unclassified_Muribaculaceae came back to roughly control levels, and both *Escherichia-Shigella* and *Enterococcus* decreased. Two other taxa, *Akkermansia* (a genus frequently linked to mucosal maintenance, although its effects appear context- and strain-dependent [36]) and Prevotellaceae_UCG-001 (a group associated with short-chain fatty acid production [37]), were more abundant in LLM@SA-treated mice than in DSS mice (Figure 4h). Consistent with the phylum-level data, LLM@SA treatment was thus associated with higher relative abundances of these taxa and a partial shift in the community toward the control profile.

**Figure 5:**
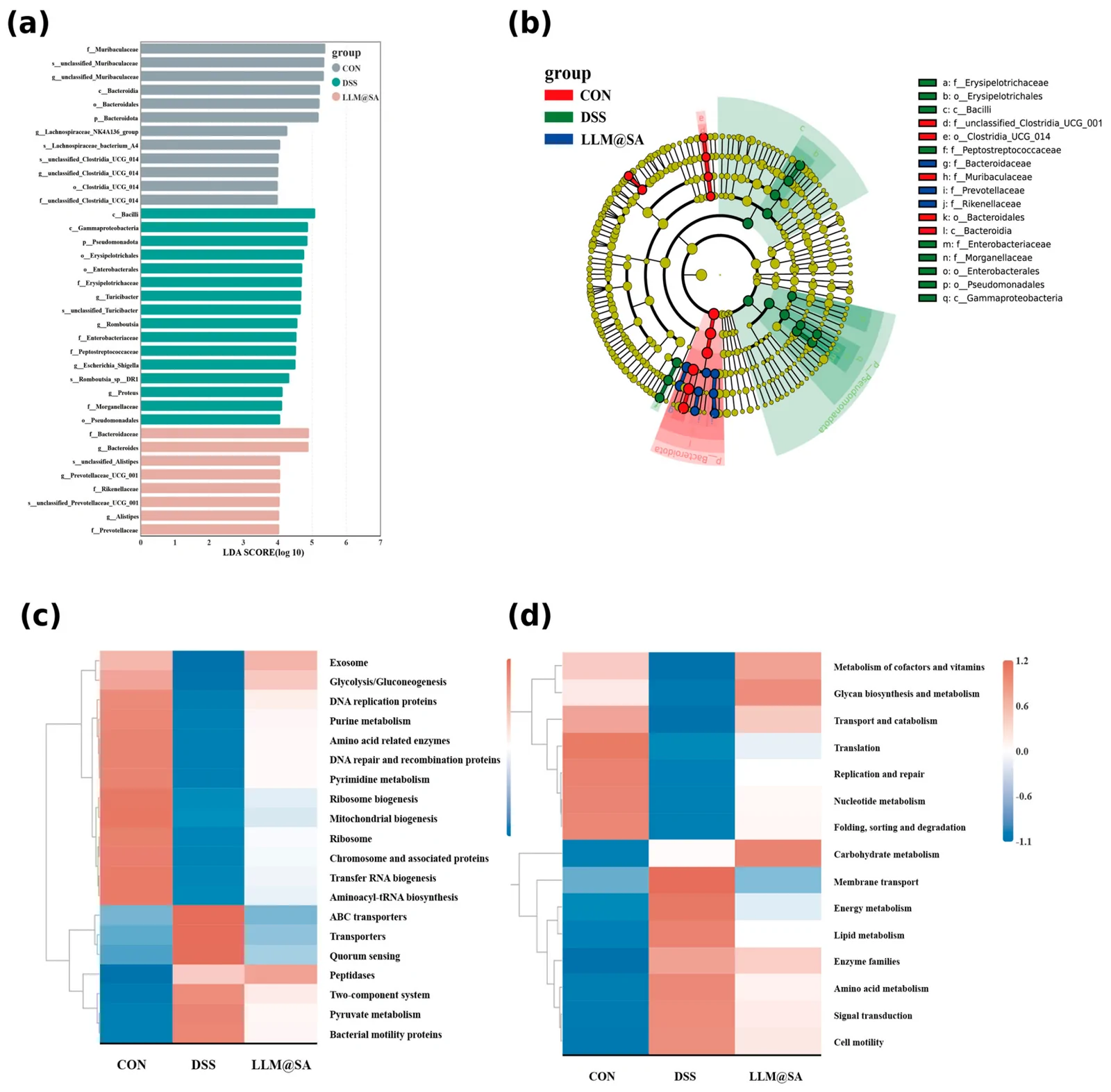
*Lactiplantibacillus plantarum* B112 microcapsules modulate gut microbial biomarkers and predicted microbial functions in DSS-induced colitis mice. (**a**) LDA score plot of differential bacterial taxa among different groups. (**b**) LEfSe cladogram of differential bacterial taxa among different groups. (**c**) Heatmap of predicted microbial functional pathways at the Kyoto Encyclopedia of Genes and Genomes (KEGG) level 3. (**d**) Heatmap of predicted microbial functional pathways at KEGG level 2.

Linear discriminant analysis effect size (LEfSe) with linear discriminant analysis (LDA) effect size estimation was applied to the fecal microbiota to identify characteristic taxa and to compare phylogenetic differences among the groups (Figure 5a,b). Distinct dominant taxa were recovered for each group, ranging from phylum to species level. The CON group was enriched in Bacteroidota, Muribaculaceae, Clostridia_UCG_014, and Lachnospiraceae_NK4A136_group; the DSS group in Pseudomonadota, Gammaproteobacteria, Enterobacterales, Enterobacteriaceae, *Proteus*, *Escherichia-Shigella*, *Turicibacter*, and *Romboutsia*, several of which include inflammation-associated or potentially opportunistic members; and the LLM@SA group in Bacteroidaceae, *Bacteroides*, Prevotellaceae, Prevotellaceae_UCG-001, Rikenellaceae, and *Alistipes*, indicating that the intervention was associated with a substantial reshaping of the DSS-disrupted microbial structure (LDA score > 2.0).

Predicted functional changes in the microbial community were examined with Tax4Fun2 (Figure 5c,d). The heatmap showed that LLM@SA attenuated the metabolic dysfunction of gut bacteria, and the LLM@SA and CON groups clustered similarly, in agreement with the compositional data. These observations suggest that LLM@SA treatment was associated with shifts in the predicted functional profile of the microbiota. We emphasize that Tax4Fun2 predictions are inferred from 16S rRNA gene profiles and therefore represent potential rather than measured microbial functions; they are strictly exploratory, and no conclusion about an actual restoration of microbial metabolic activity can be drawn without metagenomic or metabolomic confirmation.

### 3.5. Limitations and Future Directions

Because efficacy improved stepwise from LL through LLM and LLM@HA to LLM@SA, one possible interpretation is that the therapeutic effect depended on the number of viable B112 cells reaching the colon; however, CFU/g values in cecal or fecal material were not determined, so direct evidence that cell survival, and the therapeutic benefit arising from it, depended on encapsulation is still lacking. Ferric ions, alginate oligosaccharides, and gallic acid released from the MPN–polysaccharide matrix may also have played a role, but their degradation kinetics and the locally reached concentrations were not monitored, so we are for now unable to distinguish the specific contribution of each component. The design likewise did not include coating-only control groups (the MPN–polysaccharide matrix without viable B112), so the relative contributions of enhanced bacterial survival and of the encapsulation materials themselves cannot be separated. The microbiota analysis covered only four animals from each of three groups (CON, DSS, and LLM@SA); the intermediate formulations (LL, LLM, and LLM@HA) were not profiled, and cage effects were not formally modeled, so the microbial changes cannot be attributed specifically to the alginate-coated formulation as opposed to encapsulation in general or to the greater overall biological response. Nor was the statistical analysis ideal: body-weight and DAI data were compared among groups at the final time point rather than with a repeated-measures framework, community ordinations were interpreted visually without a formal multivariate test, and no correction for multiple comparisons across endpoints was applied. Finally, 16S rRNA gene amplicon sequencing detects bacterial DNA irrespective of cell viability, and sequencing of the V4 region does not achieve strain-level resolution; the sequencing data therefore cannot demonstrate that the administered B112 survived gastrointestinal transit, remained viable in the intestine, or colonized the gut. Moreover, only male mice were used as experimental subjects in this study, so possible sex-related differences were not explored. In addition, the DSS model mimics acute intestinal injury, whereas the disease course of most human IBD patients is chronic and relapsing; the adoption of chronic colitis models, larger animal species, or longer observation periods would give such work greater value for clinical translation. Because treatment was initiated only after colitis had been diagnosed, it remains unknown whether preventive administration before disease onset, or alternative treatment regimens, would prove more beneficial.

## 4. Conclusions

Wrapping *L. plantarum* B112 in a food-grade MPN–polysaccharide matrix clearly strengthened the strain’s anti-colitic effect in this DSS mouse model. Among the formulations, the alginate-coated capsules (LLM@SA) gave the broadest protection: mucosal inflammation was dampened (IL-1β, IL-6, TNF-α, MPO), IL-10 rose, epithelial barrier integrity was rebuilt (MUC2, ZO-1, occludin, LPS/D-LA/DAO), and the gut microbiota shifted toward a healthier state enriched in *Akkermansia*, *Alistipes*, and Prevotellaceae_UCG-001. A plausible mechanism, consistent with our previous in vitro protection data [14], is that the double coating improved the gastric survival of the bacteria so that more viable B112 could reach the colon; however, viable delivery was not directly measured in vivo, and a contribution of the coating materials themselves cannot be excluded. Together, the data provide preclinical proof of concept for MPN–polysaccharide microencapsulation as a food-compatible approach to probiotic delivery; conclusions regarding clinical therapy, chronic IBD, and scalability must await studies in chronic models, direct viable-count measurements, and coating-only control experiments.

## Data Availability

The raw 16S rRNA gene sequencing data have been deposited in the NCBI Sequence Read Archive (SRA) under BioProject accession number PRJNA1541253. Other data supporting the findings of this study are contained within the article and its Supplementary Material.

## Supplementary Materials

The following supporting information can be downloaded at: https://www.mdpi.com/article/10.3390/nu18203422/s1, Table S1: Disease activity index (DAI) scoring criteria for the colitis mouse model; Table S2: Histopathological scoring criteria for colon tissue; Table S3: Primer sequences for RT-qPCR of mouse colon tissue.
