# Supplementary material for: Food-Grade Metal–Phenolic Network and Polysaccharide Microencapsulation Enhances the In Vivo Anti-Colitic Efficacy of Lactiplantibacillus plantarum B112
Source: Nutrients. 2026 Oct 19;18(20):3422. doi: 10.3390/nu18203422 (PMC13708480; doi:10.3390/nu18203422)
Supplement: Supplementary file 1 [file nutrients-18-03422-s001.zip › nutrients-4572264-supplementary.pdf]

Table S1 Disease Activity Index (DAI) Scoring Criteria for the Colitis Mouse Model

Table S2 Histopathological Scoring Criteria for Colon Tissue

Table S3 Primer Sequences for RT-qPCR of Mouse Colon Tissue

Table S1 Disease Activity Index (DAI) Scoring Criteria for the Colitis Mouse Model

Score	Weight loss（%）	Stool consistency	Blood in stool
0	None	Normal	Normal
1	>0–5	Slightly loose stool	Small amount of blood in the stool
2	>5–10	Loose stool	Moderate, clearly visible blood in the stool
3	>10–15	Diarrhea	Gross bleeding
4	>15	/	/

Note: The final DAI was calculated as the sum of the weight-loss, stool-consistency, and fecal-bleeding scores (possible range: 0–12). The weight-loss intervals were revised so that each value falls unambiguously into a single category. The bleeding categories are defined by the visually detectable amount of blood in the stool; fecal blood was assessed by daily visual inspection as part of the daily DAI scoring, and no fecal occult blood test was performed.

Table S2 Histopathological Scoring Criteria for Colon Tissue

Score	Inflammation	Depth of lesion	Crypt damage	Area of lesion
0	Normal	Normal	Normal	≤1%
1	Mild	Submucosa	1/3 crypts	>1–25%
2	Moderate	Muscularis	2/3 crypts	>25–50%
3	Severe	Serosa	All, intact epithelium	>50–75%
4	Extremely severe		All, destroyed epithelium	>75%

Note: For each section, the histological score was assigned by a blinded pathologist based on a comprehensive evaluation of the four component scores listed above.

Table S3 Primer Sequences for RT-qPCR of Mouse Colon Tissue

Gene	Forward primer	Reverse primer
Gapdh	AGGTCGGTGTGAACGGATTTG	TGTAGACCATGTAGTTGAGGTCA
Occludin	ACGGACCCTGACCACTATGA	TCAGCAGCAGCCATGTACTC
MUC2	ACCTGGAAGGCCCAATCAAG	CAGCGTAGTTGGCACTCTCA
ZO-1	GGGGCCTACACTGATCAAGA	TGGAGATGAGGCTTCTGCTT
